# Impact of Heart Transplantation on Cheyne-Stokes Respiration in a Child

**DOI:** 10.1155/2016/4698756

**Published:** 2016-04-05

**Authors:** Suhail Al-Saleh, Paul F. Kantor, Indra Narang

**Affiliations:** ^1^Division of Respiratory Medicine, Hospital for Sick Children, 555 University Avenue, Toronto, ON, Canada M5G 1X8; ^2^University of Toronto, 27 King's College Cir, Toronto, ON, Canada M5S 2J7; ^3^Division of Cardiology, University of Alberta, Stollery Children's Hospital, 8440 112 Street Northwest, Edmonton, AB, Canada T6G 2B7

## Abstract

Sleep disordered breathing is well described in adults with heart failure but not in pediatric population. We describe a 13-year-old Caucasian male with severe heart failure related to dilated cardiomyopathy who demonstrated polysomnographic features of Cheyne-Stokes respiration, which completely resolved following cardiac transplantation. Cheyne-Stokes respiration in children with advanced heart failure and its resolution after heart transplant can be observed similar to adults.

## 1. Introduction

In adults with heart failure (HF), Cheyne-Stokes respiration (CSR) is found to be a common polysomnographic (PSG) finding [1]. CSR is a type of central sleep apnea (CSA) that is characterized by consecutive episodes of central apneas and/or hypopneas separated by crescendo and decrescendo breathing patterns [2]. Although CSR is secondary to HF, its presence has negative physiologic effects on cardiovascular system and it is an independent risk factor for mortality in adults with HF [1, 3, 4]. In children only two case reports have described CSR [5, 6]. We describe in this report an adolescent with dilated cardiomyopathy (DCM) who had evidence of CSR on polysomnography (PSG) which was normalized following heart transplantation.

## 2. Case Presentation

**Figure 1:**
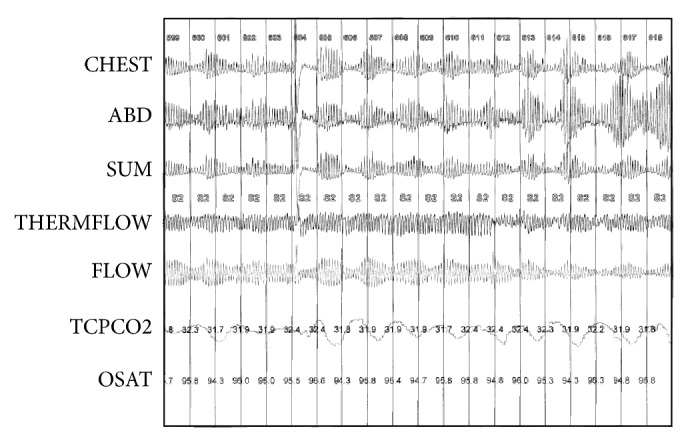
Cheyne-Stokes respiration in the child before heart transplant.

The patient, a 13-year-old Caucasian male, presented with DCM and, after progressive deterioration of his ejection fraction, eventually required admission to the hospital for progressive HF. His symptoms were increasing daytime tiredness and lethargy as well as history of syncopal episodes and increased dyspnea on effort which all were attributed to the severe heart failure. His symptoms were New York Heart Association (NYHA) class III. Specifically, echocardiography showed deterioration of his cardiac function (decreased left ventricular ejection fraction [LVEF] from 30% to 9% over a two-month period). Medical therapy had included Digoxin, Carvedilol, Ramipril, and Spironolactone. He underwent a standard PSG according to international guidelines at the time of admission that was part of a prospective observational study to describe sleep disordered breathing (SDB) in children with known cardiomyopathy, the group data of which have been published elsewhere [7]. Significant findings in the PSG were a significantly elevated central apnea-hypopnea index (CAHI) of 17 events/hour with CSR similar to that described in adults with CSR (Figure 1). He remained in the hospital for pretransplantation assessment. During this time, he was transferred to the cardiac intensive care unit and started on intravenous Milrinone.

Four weeks after the baseline PSG, and before any treatment was initiated specifically for CSR, the patient underwent an orthotropic heart transplant. Following this, he was able to recover to normal NYHA status, with no symptoms, and normal cardiac function (LVEF was 61%). A follow-up baseline PSG was done 3 months after heart transplantation to reevaluate his sleep related breathing. At this time point, he was clinically stable. His medications at this time included Prednisone, Mycophenolate Mofetil, and Tacrolimus. His repeat PSG showed that his breathing pattern had normalized with no further evidence of CSR.  Table 1 describes Echocardiographic and PSG characteristics both before and after heart transplant.

## 3. Discussion

In adults, CSR has been described in detail in patients with HF [1], with reported prevalence of CSR in patients with advanced HF of up to 37% [8]. Importantly, its presence can be a predictor of mortality [3, 4]. To our knowledge, this is the first comprehensive case report describing the presence of CSR in a child with cardiomyopathy and severely impaired cardiac function that completely resolved following heart transplantation.

Central apneas during sleep occur when PaCO_2_ falls below the apnea threshold. CSR is present when central apneas occur in cyclical fashion. The phenomenon is thought to be related to respiratory control system instability as well as a delayed circulatory time in association with low cardiac output in HF [1]. This occurs because of pulmonary vagal irritant receptor stimulation by pulmonary congestion in heart failure and increases in central and peripheral chemosensitivity [1]. The pathophysiology of respiratory control system instability leading to CSR is thought to be that of chronic hyperventilation leading to a decrease in PaCO_2_ which drops to a level close to the apnea threshold. Since patients with HF and CSR have lower PaCO_2_ than those without CSR during sleep [1], the presence of circulatory insufficiency is implicated in this pathway.

To date, there are two cases of children in HF with description of CSR [5, 6]. Hoch and Barth reported a 6-month-old boy with trisomy 21 and repaired atrioventricular septal defect who had pulmonary hypertension presented, had increased lethargy, and had CSR features on PSG. Rao and Arens posted a case report online [6] with clinical sleep fragments of a 16-year-old obese boy with DCM and congestive heart failure requiring inotropic support. This patient had PSG features of CSR. Our case supports these findings, confirming the presence of CSR in association with severely impaired cardiac function, and further confirms the complete resolution of the sleep disordered breathing following heart transplantation.

In children, cardiomyopathy is a rare but serious and life-threatening condition with an annual incidence of 1.1–1.2/100,000. Dilated cardiomyopathy is the dominant phenotype, with two-thirds of all cases remaining idiopathic after diagnostic evaluation. The prognosis of DCM in children is guarded with increased morbidity and mortality, and currently 32–46% of patients with DCM will either receive a heart transplant or die within five years of diagnosis. Our patient had severe DCM with severely impaired cardiac function and he received an urgent heart transplant because of his deteriorating condition.

Several adult case reports and series have shown resolution and improvement of CSR in patients with HF after heart transplant [9], occurring as early as 9 weeks after a heart transplant [10]. However, a proportion of patients will have persistent CSR or convert to obstructive sleep apnea despite normalization of cardiac function, for unclear reasons [9, 10], but it has been suggested that this could be related to increased weight after heart transplant [11]. The resolution of CSR features in our patient supports the concept that the phenomenon is typically driven by circulatory insufficiency in children, as in adults.

In conclusion CSR can be present in children with advanced heart failure. We speculate that it may contribute to the symptom progression in these patients and may be a risk factor for death for that reason, although there are insufficient cases to determine this at present. Resolution of CSR after pediatric heart transplant would be expected assuming no other contributory factors are identified. Prospective evaluation of patients for the presence of sleep related disordered breathing appears justified, even in the pediatric age group, when advanced heart failure is identified.

## Figures and Tables

**Table 1:** PSG results of the patient before and after heart transplant.

Results	Pretransplant	Posttransplant
TST, min	358.5	389.5
Sleep latency, min	10.8	1.8
Sleep efficiency, %	96.5	91.5
REM latency, min	159	167
Stage 1% TST	0.6	1.4
Stage 2% TST	59.6	54
Slow wave sleep % TST	21.2	24.9
REM % TST	18.7	19.6
Arousals, total index	32.8	10
Mean sleep SaO_2_ (%)	93	98
Minimum SaO_2_ (%)	85	94
Highest TcCO_2_/etCO_2_ (mmHg)	35	45
OAHI (events/hour)	1.2	0.5
CAHI (events/hour)	17.6	0.9
LVEF% (Simpsons)	9	61

CAHI: central apnea-hypopnea index, etCO_2_: end tidal carbon dioxide, LVEDd: left ventricular diastolic dimension, LVEF: left ventricular ejection fraction, OAHI: obstructive apnea-hypopnea index, REM: rapid eye movements, SaO_2_: oxygen saturation, TcCO_2_: transcutaneous carbon dioxide, and TST: total sleep time.

## Abbreviations

CSA: Central sleep apnea
CAI: Central apnea index
CSR: Cheyne-Stokes respiration
DCM: Dilated cardiomyopathy
HF: Heart failure
LVEF: Left ventricular ejection fraction
OSA: Obstructive sleep apnea
OAHI: Obstructive apnea-hypopnea index
PSG: Polysomnography
SDB: Sleep disordered breathing.
